# Navigating the Needles: Unveiling the Risks of Acupuncture: A Case Report

**DOI:** 10.1002/ccr3.9637

**Published:** 2024-11-27

**Authors:** Harkesh Arora, Dhiran Sivasubramanian, Sinduja Sivakumar, Sivaguha Yadunath, Sreekant Avula

**Affiliations:** ^1^ Lovelace Medical Center Albuquerque New Mexico USA; ^2^ Christian Medical College and Hospital Vellore Vellore India; ^3^ Burrell College of Osteopathic Medicine Las Cruces New Mexico USA; ^4^ Mercy Catholic Medical Center—Mercy Fitzgerald Campus Darby Pennsylvania USA; ^5^ University of Minnesota Minneapolis Minnesota USA

**Keywords:** acupuncture, alternative medical therapy, complications, pneumothorax

## Abstract

Acupuncture, an increasingly popular alternative medical therapy, has heightened the demand for information on its safety profile. This is a case report involving a 28‐year‐old female who presented with intense right‐sided chest pain and breathlessness after an acupuncture session where needles were inserted into her chest wall. The immediate medical evaluation revealed a tension pneumothorax with radiographic evidence of a mediastinal shift, compelling, urgent intervention via chest tube insertion. The patient's symptoms persisted for 2 days before the pneumothorax diminished, facilitating the removal of the chest tube. This case emphasizes the atypical presentation of pneumothorax after acupuncture, suggesting a delayed onset of symptoms possibly related to the size of the bronchopleural fistula formed by the acupuncture needle. While acupuncture‐related adverse events are uncommon, serious life‐threatening complications like tension pneumothorax underscore the necessity for rigorous safety standards. Amidst the growing popularity of acupuncture, this report urges healthcare professionals to be aware of and report such incidents for enhanced patient education and safety.

**Trial Registration:** This is not an interventional study. We only reported the patient's findings from our database as a case report

**Summary**

- Acupuncture as an alternative medical therapy has grown in popularity in recent years, prompting a surge in demand for reliable safety information.
- While acupuncture‐related complications are generally rare, they can cause minor side effects to severe consequences like infection transmission and, in extreme cases, tension pneumothorax.
- Awareness and reporting of such complications are crucial for patient education and ensuring the competence of practitioners.

## 1 Introduction

Acupuncture is an alternative medical therapy that has grown in popularity. Acupuncture's widespread use has increased the demand for reliable information on safety concerns [1]. Acupuncture‐related complications are uncommon, but they range from minor side effects like local pain and hemorrhage to more serious complications like infection transmission (hepatitis B and C, HIV infection), pneumothorax, cardiac tamponade, vascular lesions, and spinal cord injuries [2].

Postmortem examinations revealed that a 10–20 mm deep puncture is sufficient to reach the lung because its surface is about 15–20 mm beneath the skin [3]. Acupuncture complications are more commonly reported by those who manage them than by the acupuncturist [2]. Only 20% of the reports were written by the acupuncturist who performed the procedure that caused the adverse effect [4].

With the widespread use of acupuncture, there is a greater need for reliable information on safety concerns. Because acupuncture is a growing alternative medical therapy, raising awareness of the potential complications of this invasive procedure is essential. Reporting acupuncture‐related complications can help with patient education, awareness, and the need for an accredited and registered professional. We present a case of tension pneumothorax following acupuncture, which is a potentially fatal complication.

## 2 Case History and Examination

A 28‐year‐old female presented with complaints of severe right‐sided chest pain following acupuncture in which needles were inserted into the right chest wall. Even though multiple attempts were made, the patient could not describe the details of the acupuncture procedure that was performed at an outside smaller office location. When the patient began to experience shortness of breath, she decided to go to the bigger emergency room (ER) with trauma capabilities. The pain began spontaneously and was pleuritic, with an intensity rating of 8 out of 10. The right‐sided chest pain began 30 min before the ER visit and radiated to her shoulder. Deep breaths, movement, and coughing aggravated the pain. In addition, the patient had history of anxiety and depression. The patient did not smoke. She was taking sertraline for depression. She was afebrile, blood pressure was 126/82 mmHg, heart rate was 88 beats per minute, and her oxygen saturation was 94% on room air.

### 2.1 Investigations and Treatment

**FIGURE 1:**
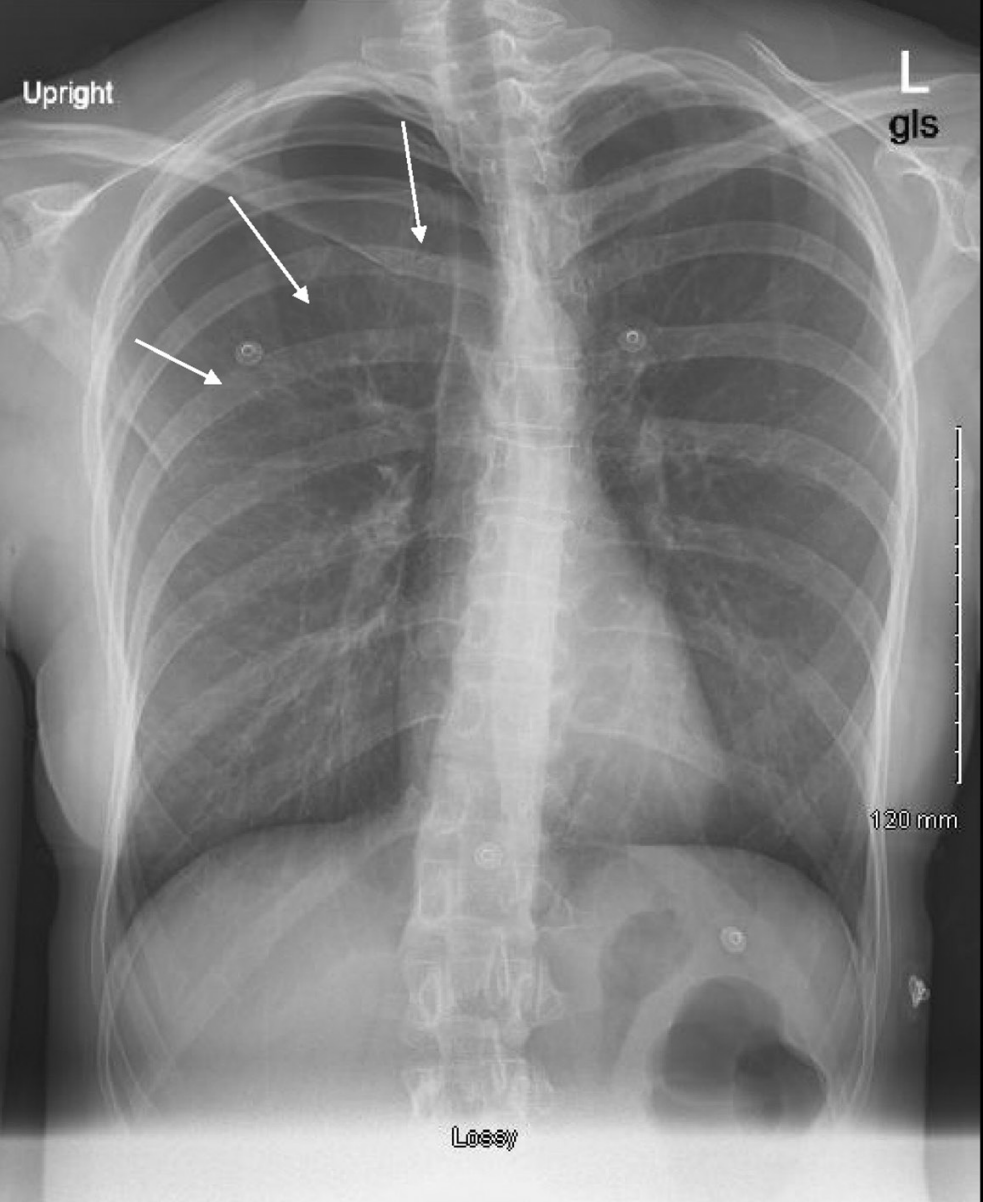
Right‐sided pneumothorax noted in the apical region with mediastinal shift to the left.

**FIGURE 2:**
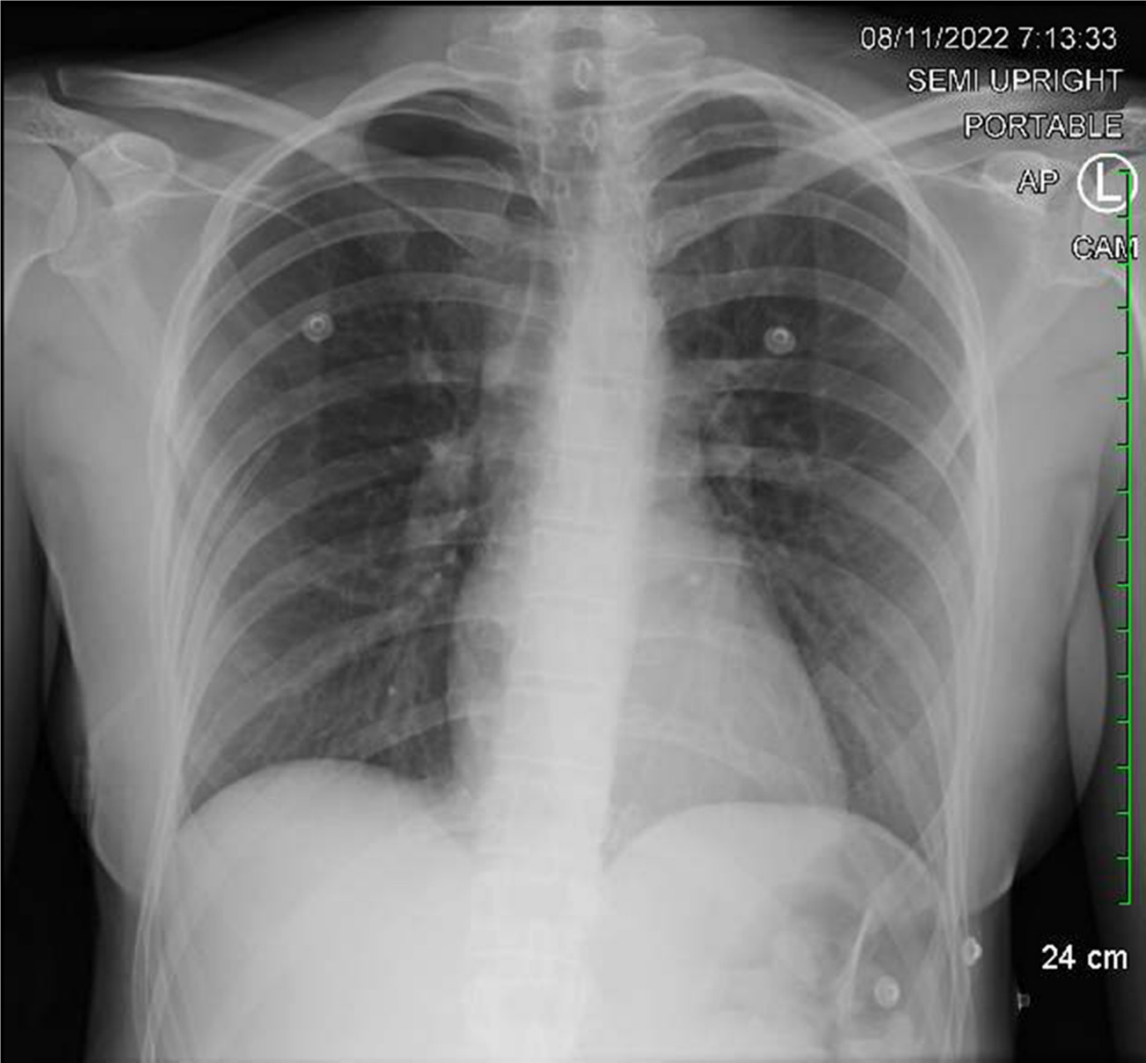
Right‐sided pneumothorax noted in the apical region with mediastinal shift to the left with chest tube.

Soon after the physical examination, the patient was started on nasal cannula oxygen therapy. The first chest x‐ray revealed a pneumothorax with no shifting. Due to worsening shortness of breath, a follow‐up x‐ray revealed a right‐sided pneumothorax in the apical region, 40.5% measuring 38 mm, with a mediastinal shift to the left which was concerning for the development of tension pneumothorax (Figure 1). As a result, a right chest tube was immediately inserted by the ER physician and pulmonology team was consulted who managed the patient (Figure 2). Hemoglobin was 11.5 g/dL, WBC was 6.2 K/μL, and platelets were 146 K/μL. The sodium concentration was 142 mmol/L, the potassium concentration was 4.0 mmol/L, the chloride concentration was 110 mmol/L, and the calcium concentration was 8.4 mg/dL. Arterial Blood Gas (ABG) was not drawn as the patient remained stable and room air oxygen saturation level was appropriate. After remaining persistent for 2 days, the pneumothorax shrunk to 20 mm. The chest tube was removed, and the patient appeared comfortable on room air. She was finally discharged home with instructions to resume home medications.

## 3 Conclusion

Complications are sure to develop as alternative types of medicine become more popular. Pneumothorax is a potentially fatal consequence of acupuncture that should be investigated in any patients who develop shortness of breath after treatment.

## 4 Discussion

Acupuncture is a widely recognized alternative therapy, though evidence supporting its efficacy is inconclusive, mainly limited to treating back and neck pain, assisted conception, idiopathic headaches, and chemotherapy‐related nausea [5]. Reported complications include pneumothorax [6], pericardial effusion [7], nerve injury [8], and trauma [9]. A 2022 case series highlighted pneumothorax incidents, noting patients were often uninformed of risks [10]. Another study identified pneumothorax, fainting, subarachnoid hemorrhage, and infection as common adverse effects associated with acupuncture [11].

Our case aligns with existing literature on acupuncture‐related pneumothorax. While adverse effects are rare, serious complications require vigilance. The case series on pneumothorax emphasizes that patient unawareness of risks can delay treatment [10], which aligns with our findings, where prompt management was key in preventing severe outcomes like tension pneumothorax.

While some risk is acceptable with evidence‐based acupuncture, our patient's severe respiratory distress, worsened by tension pneumothorax and mediastinal shift, underscores the variability in outcomes based on patient factors and practitioner skill and experience. This case highlights the importance of skilled administration and awareness of potential complications.

The sequence of events in our case suggests a direct link between acupuncture and pneumothorax, with symptoms delayed, possibly due to a small bronchopleural fistula formed by the acupuncture needle. Although the initial X‐ray did not show a large pneumothorax, the patient's condition worsened due to a malfunctioning ball valve mechanism, posing an immediate threat to life [12]. A chest tube was used which are effective but pigtail catheters, which offer simpler installation and fewer complications and a shorter hospital stay, could have been considered [13].

Given the risk of serious complications, acupuncture should be performed by trained professionals capable of recognizing and managing adverse events. Involving specialists like anesthetists, intensivists, and thoracic surgeons and performing acupuncture in well‐equipped centers with emergency resources can improve patient safety. Additionally, educating patients on risks and benefits, and standardizing acupuncture practices, can also help minimize risks and improve outcomes supporting a safer, more holistic approach to pain management.

As acupuncture becomes more common, the risk of complications will increase. Although serious adverse events are rare—0.05 per 10,000 treatments—pneumothorax remains the most common and can be fatal [14]. These events are often preventable through proper training, technique, and sterilization. Establishing strict safety standards is crucial to reduce risks greatly and enhance patient safety [15].

In conclusion, while acupuncture is an effective alternative therapy, addressing its risks and ensuring high standards of practice are essential. Emphasizing patient education, professional training, and emergency preparedness can improve acupuncture's safety and efficacy in clinical settings.

## Author Contributions

**Harkesh Arora:** conceptualization, formal analysis, resources, software, supervision, writing – original draft, writing – review and editing. **Dhiran Sivasubramanian:** conceptualization, writing – original draft, writing – review and editing. **Sinduja Sivakumar:** resources, writing – review and editing. **Sivaguha Yadunath:** resources, writing – review and editing. **Sreekant Avula:** funding acquisition, resources, writing – review and editing.

## Ethics Statement

This is a case report that does not require formal ethical committee approval. The data were anonymously registered in our database.

## Consent

Written informed consent was obtained from the patient to publish this report in accordance with the journal's patient consent policy. A copy of the written consent is available for review by the Editor‐in‐Chief of this journal on request.

## Conflicts of Interest

The authors declare no conflicts of interest.

## Data Availability

Data sharing is not applicable to this article as no new data were created or analyzed in this study.

## References

[1] M. Stenger , N. E. Bauer , and P. B. Licht , “Is Pneumothorax After Acupuncture So Uncommon?,” Journal of Thoracic Disease 5, no. 4 (2013): E144, 10.3978/j.issn.2072-1439.2013.08.18. 23991325 PMC3755677

[2] S. C. Corado , M. Graça Santos , L. Quaresma , and J. R. Baltazar , “Pneumothorax After Acupuncture,” British Medical Journal Case Reports 12, no. 6 (2019): e228770, 10.1136/bcr-2018-228770. PMC6578517 31189543

[3] E. T. Peuker , A. White , E. Ernst , F. Pera , and T. J. Filler , “Traumatic Complications of Acupuncture. Therapists Need to Know Human Anatomy,” Archives of Family Medicine 8, no. 6 (1999): 553, 10.1001/archfami.8.6.553. 10575398

[4] E. Ernst and K. J. Sherman , “Is Acupuncture a Risk Factor for Hepatitis? Systematic Review of Epidemiological Studies,” Journal of Gastroenterology and Hepatology 18, no. 11 (2003): 1231–1236, 10.1046/j.1440-1746.2003.03135.x. 14535978

[5] E. Manheimer , S. Wieland , E. Kimbrough , K. Cheng , and B. M. Berman , “Evidence From the Cochrane Collaboration for Traditional Chinese Medicine Therapies,” Journal of Alternative and Complementary Medicine 15, no. 9 (2009): 1001–1014, 10.1089/acm.2008.0414. 19757977 PMC2856612

[6] D. A. Hampton , R. T. Kaneko , E. Simeon , A. Moren , S. Rowell , and J. M. Watters , “Acupuncture‐Related Pneumothorax,” Acupuncture in Medicine 26, no. 4 (2014): 241–245, 10.1089/acu.2013.1022. PMC4142775 25184016

[7] X. F. Li and X. Wang , “Acupuncture Therapy Related Cardiac Injury,” Chinese Journal of Integrative Medicine 19, no. 12 (2013): 885–888, 10.1007/s11655-013-1355-4. 23739993

[8] C. L. Lin , A. Chern , M. J. Wang , and S. K. Lin , “Incidence of Nerve Injury Following Acupuncture Treatments in Taiwan,” Complementary Therapies in Medicine 80 (2024): 103007, 10.1016/j.ctim.2023.103007. 38040097

[9] T. E. Kao , Y. W. Kuo , and K. Y. Wu , “Acupuncture‐Related Penetrating Eye Injury,” Kaohsiung Journal of Medical Sciences 33, no. 9 (2017): 473–474, 10.1016/j.kjms.2017.05.006.28865606

[10] F. Th'ng , K. A. Rao , and P. Y. Huang , “Case Series: Acupuncture‐Related Pneumothorax,” International Journal of Emergency Medicine 15, no. 1 (2022): 48, 10.1186/s12245-022-00455-z. 36096724 PMC9465868

[11] J. Zhang , H. Shang , X. Gao , and E. Ernst , “Acupuncture‐Related Adverse Events: A Systematic Review of the Chinese Literature,” Bulletin of the World Health Organization 88, no. 12 (2010): 915–921C, 10.2471/BLT.10.076737. 21124716 PMC2995190

[12] W. I. Choi , “Pneumothorax,” Tuberculosis Respiratory Disease 76, no. 3 (2014): 99–104, 10.4046/trd.2014.76.3.99. PMC3982243 24734096

[13] S. H. Chang , Y. N. Kang , H. Y. Chiu , and Y. H. Chiu , “A Systematic Review and Meta‐Analysis Comparing Pigtail Catheter and Chest Tube as the Initial Treatment for Pneumothorax,” Chest 153, no. 5 (2018): 1201–1212, 10.1016/j.chest.2018.01.048. 29452099

[14] A. White , “A Cumulative Review of the Range and Incidence of Significant Adverse Events Associated With Acupuncture,” Acupuncture in Medicine 22, no. 3 (2004): 122–133, 10.1136/aim.22.3.122. 15551936

[15] W. He , X. Zhao , Y. Li , Q. Xi , and Y. Guo , “Adverse Events Following Acupuncture: A Systematic Review of the Chinese Literature for the Years 1956‐2010,” Journal of Alternative and Complementary Medicine 18, no. 10 (2012): 892–901, 10.1089/acm.2011.0825. 22967282

